# Sex differences in toxicity and outcomes in patients with sarcoma treated in the perioperative setting at a comprehensive cancer center

**DOI:** 10.3389/fonc.2025.1585884

**Published:** 2025-06-26

**Authors:** Ilaria Tortorelli, Benedetta Chiusole, Fabio Murtas, Antonella Galiano, Maital Bolshinsky, Selma Ahcene-Djaballah, Chiara De Toni, Salvatore Vizzaccaro, Marco Maruzzo, Umberto Basso, Alberto Banzato, Marina Coppola, Sara Lonardi, Vittorina Zagonel, Antonella Brunello

**Affiliations:** ^1^ Oncology 1 Unit, Department of Oncology, Veneto Institute of Oncology IOV - Istituto di Ricovero e Cura a Carattere Scientifico (IRCCS), Padua, Italy; ^2^ Department of Surgery, Oncology and Gastroenterology (DISCOG), University of Padua, Padua, Italy; ^3^ Oncology 3 Unit, Department of Oncology, Veneto Institute of Oncology IOV - Istituto di Ricovero e Cura a Carattere Scientifico (IRCCS), Padua, Italy; ^4^ Cardiology Unit, Veneto Institute of Oncology IOV - Istituto di Ricovero e Cura a Carattere Scientifico (IRCCS), Padua, Italy; ^5^ Pharmacy Unit, Veneto Institute of Oncology IOV - Istituto di Ricovero e Cura a Carattere Scientifico (IRCCS), Padua, Italy

**Keywords:** sarcoma, toxicity, outcome, sex differences, relative dose intensity

## Abstract

**Background:**

There is an unmet need of personalized strategies taking into account the influence of sex on treatment. Toxicities commonly lead to dose reductions or delays, which may impact outcomes. The current retrospective study investigated the impact of sex on chemotherapy efficacy and toxicity, and evaluated the effect of Relative Dose Intensity (RDI) on survival in patients with sarcoma.

**Material and methods:**

Data of patients with localized high-grade sarcoma treated at the Veneto Institute of Oncology – IRCCS between 2010 and 2022 were analyzed. Dose reduction or delay were expressed as RDI. Sex differences in RDI, severe adverse events (AEs) and the impact of RDI on disease-free survival and overall survival were analyzed.

**Results:**

A total of 215 patients (women, 46.5%; men, 53.5%) were eligible. Of these, 127 patients were affected by high-grade soft-tissue sarcoma and treated with anthracycline-based chemotherapy. Males were more likely to receive RDI ≥85%, with a lower risk of AEs compared to females. An RDI ≥85 was associated with improved survival outcomes.

**Conclusions:**

To the authors’ knowledge, this is the first study investigating the impact of sex on toxicity and efficacy of perioperative chemotherapy in patients with sarcomas. The increased toxicity in women suggests there is a sex difference in treatment delivery and outcome. Despite a lower RDI, survival outcomes for women were not worse than men. Future studies should aim to better optimize drug dosing according to the sex, with the ultimate goal of increasing therapeutic benefit while limiting toxicity.

## 1 Introduction

The incidence and severity of a wide range of tumors are influenced by differences between males and females. Epidemiologic studies underscore sex differences in susceptibility and survival of non-sex-related cancer (1). With the exception of thyroid cancer, incidence and mortality of non-reproductive tumors is higher in males than in females (2, 3). In 2016, Clocchiatti and colleagues introduced the term “sexual dimorphism” to describe sex-related differences in cancer (4). After the National Institutes of Health (NIH) proposed to consider sex as a biological variable, several researchers started to pay more attention to the molecular mechanisms behind sex differences in cancer (5). Beyond the role of gonadal hormones, sex differences are thought to be due to genetic and molecular pathways involved in cancer susceptibility and proliferation, as well as in treatment response (6). Sex differences have been observed in terms of efficacy and toxicity of conventional chemotherapy, as well as targeted therapies and immunotherapy (4–7). In this context, it is important to consider that historically, women have often been excluded from clinical trials for non-sex-related cancers. In 1977, the United States Food and Drug Administration (FDA) issued a guidance document “General Considerations for the Clinical Evaluation of Drugs”, advising that women of childbearing potential should be excluded from early phase clinical research, with the exception of trials testing drugs for life-threatening illness (8). It was not until 1993, after the National Institutes of Health (NIH) had established a policy on the inclusion of women in clinical trials, that the FDA reversed the 1977 guidance (2). In 2000, the FDA issued a final rule that has given the authority to place a clinical hold on a trial for a life-threatening disease if sponsors exclude men or women solely on the basis of reproductive potential (9). Although today women are systematically included in trials and despite growing evidence of the role of sex in treatment personalization, patient sex is rarely taken into account in clinical research. Furthermore, although most dosing regimens are based on patient-specific parameters (body surface area or body weight), chemotherapy is often complicated by severe toxicity, which often require dose reduction or delay of planned treatment (10). Relative dose intensity is defined as the ratio of the delivered dose intensity (dose per unit body surface area per unit time [mg/m2 per week]) to the standard or planned dose intensity (10). It is a summary measure commonly used to describe dose reductions and/or treatment delays that occur with a chemotherapy regimen (10–12). Clinical evidence suggests that the dose intensity of chemotherapy is an important predictor of clinical outcome (13–17). This has been observed primarily in studies of early stage breast cancer (15, 16), but it has been proven to hold true in several other solid cancers (18). The Norton-Simon hypothesis suggests that more frequent administration of chemotherapy can reduce residual tumor burden, while according to the Goldie-Coldman hypothesis, high-intensity dose regimens appear to prevent the accumulation of mutations that could lead to drug resistance (14, 19). The role of dose intensity is of particular relevance in some sarcomas, particularly Ewing sarcoma, in which it has been shown that interval-compressed chemotherapy (every two instead of every three weeks) carries superior outcomes (20). Dose intensification has not been associated with significantly improved outcomes in osteosarcoma or soft tissue sarcomas (21, 22). In this scenario we retrospectively analyzed data from patients treated for sarcomas at a Comprehensive Cancer Center to investigate the influence of sex on severe acute toxicity and outcomes. In particular, we sought to understand whether female sex was associated with a higher risk of adverse events from perioperative chemotherapy and how treatment-related toxicities led to dose reductions and/or therapy delays, thus affecting patient survival.

## 2 Patients and methods

This is a retrospective observational study conducted at Veneto Institute of Oncology (IOV) – IRCCS, Padua. Data of consecutive adult patients with localized high-grade sarcoma treated between 2010 and 2022 were retrieved from a prospectively maintained database. Inclusion criteria were: adult (>18 years) patients with diagnosis of either bone or high-grade soft tissue sarcoma, localized stage of disease with indication to either neoadjuvant or adjuvant chemotherapy. Patients receiving first-line treatment for unresectable and/or metastatic disease were excluded, as well as patients with diagnosis of gastrointestinal stromal tumors (GIST) and patients with a history of other malignancies unless in remission for 5 years or more. Patients for whom sufficient data were not available for the analyses were also excluded. Information on patient age and sex, performance status, treatment, adverse events, laboratory results, outcomes, and tumor characteristics were collected. The study was approved by IOV Ethics Committee. For the sake of the analysis, the different histologic subtypes were aggregated into ten common groups: angiosarcoma (AS), chondrosarcoma (CS), leiomyosarcoma (LMS), liposarcoma (LPS), malignant peripheral nerve sheath tumor (MPNST), synovial sarcoma (SS), undifferentiated pleomorphic sarcoma (UPS), osteosarcoma (OS), Ewing sarcoma (ES) and a group named ‘Other’, containing all other histotypes. Chemotherapy regimens were grouped into six main categories: ‘anthracycline-based doublet’ (e.g., epirubicin plus ifosfamide or doxorubicin plus dacarbazine), ‘anthracycline monotherapy’ (e.g., doxorubicin alone), ‘gemcitabine-based doublet’ (e.g., gemcitabine plus dacarbazine or gemcitabine plus docetaxel), ‘monotherapy’ (e.g., ifosfamide/paclitaxel/gemcitabine/trabectedin), ‘osteosarcoma/Ewing-like therapy’ (regimens including doxorubicin, cisplatin, high-dose methotrexate, ifosfamide/cyclophosphamide, vincristine, dactinomycin and etoposide) and a group named ‘Other’, containing all the other chemotherapy regimens. In addition to the data analysis of the general population of patients with localized high-grade sarcoma (Group 1) - in order to reduce regimens-related bias - data of patients affected by soft tissue sarcoma treated with anthracycline-based chemotherapy were then separately analyzed in a subgroup analysis (Group 2). Dose reductions and/or delays were evaluated during the first 9 weeks of chemotherapy and expressed as RDI. In line with available literature, a reduction in RDI below 85% was considered to be a clinically significant reduction from standard or planned therapy. For patients receiving multi-agent chemotherapy, RDI was calculated as the mean of the RDI for each agent. To establish a common reference, all adverse events (AE) codes and grades were mapped to Version 5 of the Common Terminology Criteria for Adverse Events (CTCAE) (23). On the basis of observed patterns, AEs were categorized as hematologic (anemia, thrombocytopenia, afebrile/febrile neutropenia, lymphocytopenia) or non-hematologic (liver or renal alterations, dysuria/strangury, non-infectious cystitis, hematuria, diarrhea/constipation, nausea/vomiting, mucositis/toothache, asthenia, edema, cutaneous toxicity, central/peripheral neurologic toxicity, influenza-like symptoms, infusion reaction, cardiovascular disorders, urinary infection, eye disorders). The CTCAE are graded from 0 to 5, where 0 indicates no toxicity; 1, mild; 2, moderate; 3, severe; 4, life-threatening; and 5, death (23). AEs of unknown grade and sex-specific AEs (male and female sexual function) were excluded. The primary objective was to assess whether severe acute toxicity and RDI levels differ between male and female patients treated with chemotherapy for localized sarcoma. Secondary objective was to evaluate the impact of sex and RDI on survival outcomes in terms of overall survival (OS) and disease-free survival (DFS).

### 2.1 Statistical methods

Descriptive analyses were used to examine patients’ characteristics and clinical outcomes. Comparisons were made using the Chi-squared test, Fisher’s exact test or Wilcoxon rank-sum test, as appropriate. The Wilcoxon rank-sum test was used to compare males and females for age at diagnosis, as the data were not normally distributed. Treatment, primitive and body mass index (BMI) were analysed with Chi-squared test; while Fisher’s exact test was applied for the variables diagnosis and chemotherapy, as they showed low frequencies. Survival analyses were performed using the Kaplan-Meier method and the log-rank test was used to compare survival curves. Median follow-up was calculated using the reverse Kaplan-Meier method. Disease-free survival (DFS) was calculated as the time from the date of therapy initiation to the date of cancer recurrence or to the last follow-up. Overall survival (OS) was defined as the time from treatment initiation to death from any cause or to the last follow-up. Univariable and multivariable logistic regression models were performed to test the association between RDI or G3-G4 toxicity and the exploratory variables: sex, age, chemotherapy regimen, body mass index (BMI). Area under the curve (AUC) was used to assess the goodness of fit of the multivariable models. Analyses were performed in September 2023 using the R software, version 4.3.1. The significance level was set at 5%.

## 3 Results

### 3.1 Patient characteristics

**Figure 1:**
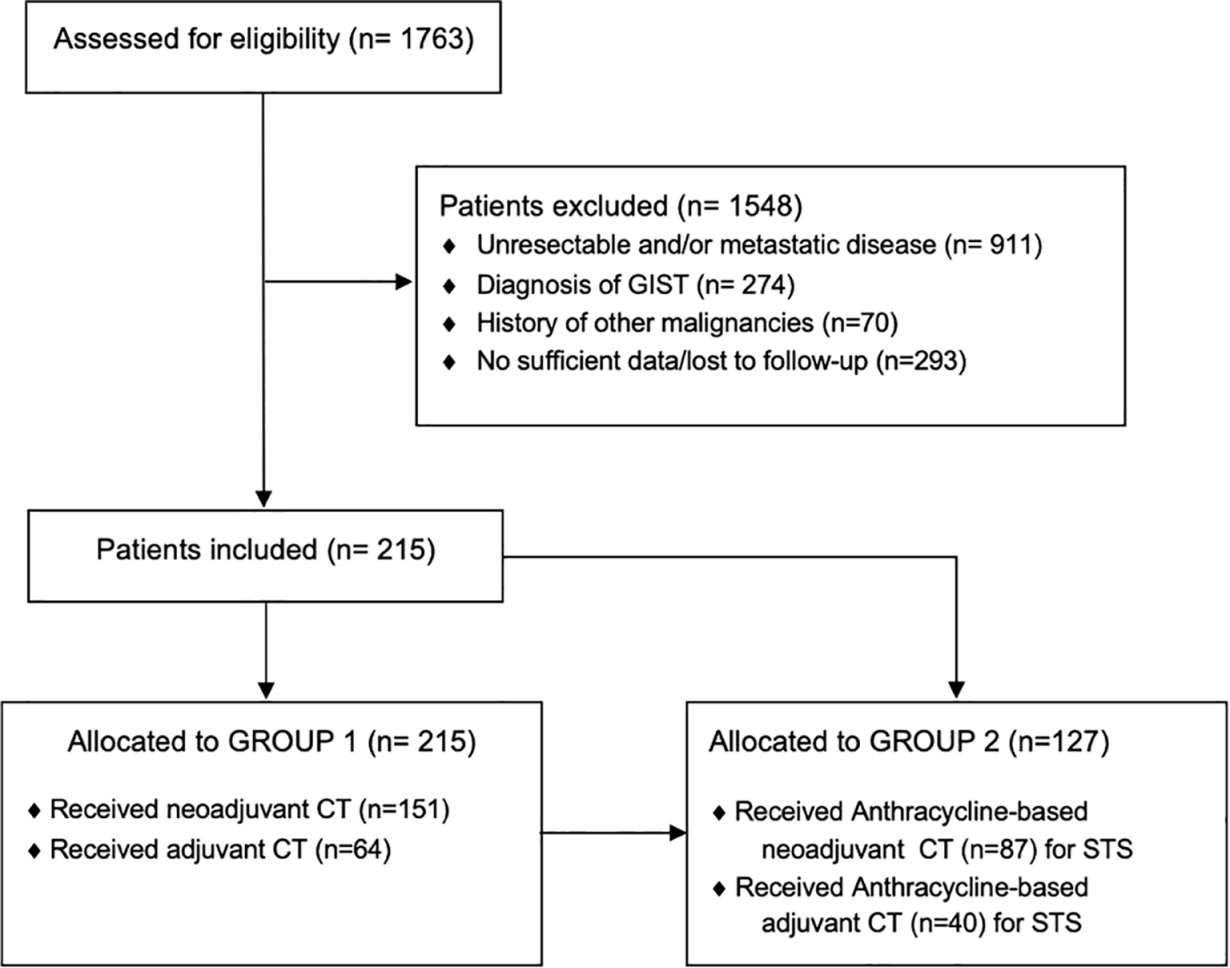
STROBE diagram. STROBE, Strengthening the reporting of observational studies in epidemiology; GIST, Gastrointestinal stromal tumor; CT, Chemotherapy.

In total, we analyzed data from 215 patients (Group 1: women, 100 [46.51%]; men, 115 [53.49%]) treated with neoadjuvant (151 [70.23%]) or adjuvant (64 [29.77%]) chemotherapy for localized high-grade sarcoma (**Figure 1**). Of these, a sub-group of 127 patients (Group 2) was affected by high-grade soft tissue sarcomas and was treated with anthracycline-based perioperative chemotherapy (women, 54 [42.52%]; men, 73 [57.48%]), with 87 (68.5%) patients treated in the neoadjuvant setting and 40 (31.5%) in the adjuvant setting. Patients’ characteristics are summarized in **Tables 1** and **2**.

**Table 1:** Patients’ characteristics for group 1 (n=215).

Variables	Total 215 (100)	Female 100 (46.51)	Male 115 (53.49)	p-value
Median age at diagnosis (IQR)	52.00 (40.00-63.50)	53.00 (41.00-63.25)	51.00 (40.00-63.50)	0.6609
<65 years	168 (78.14)	78 (78.00)	90 (78.26)	1
≥65 years	47 (21.86)	22 (22.00)	25 (21.74)	
Treatment				
Neo-adjuvant	151 (70.23)	67 (67.00)	84 (73.04)	0.4138
Adjuvant	64 (29.77)	33 (33.00)	31 (26.96)	
Primitive				
Bone	39 (18.14)	19 (19.00)	20 (17.39)	0.8982
Soft tissue	176 (81.86)	81 (81.00)	95 (82.61)	
Diagnosis				
Angiosarcoma	19 (8.84)	11 (11.00)	8 (6.96)	0.5692
Chondrosarcoma	9 (4.19)	4 (4.00)	5 (4.35)	
Leiomyosarcoma	25 (11.63)	14 (14.00)	11 (9.56)	
Liposarcoma	43 (20.00)	18 (18.00)	25 (21.74)	
Mpnst	3 (1.40)	0 (0.00)	3 (2.61)	
Osteosarcoma	22 (10.23)	11 (11.00)	11 (9.56)	
Other	36 (16.74)	16 (16.00)	20 (17.39)	
Ewing sarcoma	14 (6.51)	4 (4.00)	10 (8.70)	
Synovial sarcoma	11 (5.12)	7 (7.00)	4 (3.48)	
Ups	33 (15.35)	15 (15.00)	18 (15.65)	
Chemotherapy				
Anthracycline-based doublet	125 (58.14)	56 (56.00)	69 (60.00)	**0.0369**
Anthracycline monotherapy	5 (2.32)	0 (0.00)	5 (4.35)	
Gemcitabine-based doublet	7 (3.26)	6 (6.00)	1 (0.87)	
Monotherapy (ifosfamide/paclitaxel/gemcitabine/trabectidine)	31 (14.42)	16 (16.00)	15 (13.04)	
Osteosarcoma/Ewing-like	45 (20.93)	20 (20.00)	25 (21.94)	
Other	2 (0.93)	2 (2.00)	0 (0.00)	
BMI				
<25	117 (54.42)	62 (62.00)	55 (47.83)	**0.0519**
≥25	98 (45.58)	38 (38.00)	60 (52.17)	

MPNST, malignant peripheral nerve sheath tumor; UPS, undifferentiated pleomorphic sarcoma; IQR, interquartile range; BMI, body mass index.

The bold values refer to those that are statistically significant.

**Table 2:** Patient characteristics for group 2 (n=127).

Variables	Total 127 (100)	Female 54 (42.52)	Male 73 (57.48)	p-value
Median age at diagnosis (IQR)	52.00 (43.00-62.25)	52.50 (42.25-62.75)	52.00 (43.00-62.00)	0.8243
<65 years	103 (81.10)	44 (81.48)	59 (80.82)	1
≥65 years	24 (18.90)	10 (18.52)	14 (19.18)	
Treatment				
Neo-adjuvant	87 (68.50)	36 (66.67)	51 (69.86)	0.8492
Adjuvant	40 (31.50)	18 (33.33)	22 (30.14)	
Diagnosis				
Angiosarcoma	3 (2.36)	3 (5.56)	0 (0.00)	0.4338
Extra-skeletal Myxoid Chondrosarcoma	1 (0.79)	0 (0.00)	1 (1.37)	
Leiomyosarcoma	16 (12.60)	6 (11.11)	10 (13.70)	
Liposarcoma	35 (27.56)	14 (25.93)	21 (28.76)	
Mpnst	3 (2.36)	0 (0.00)	3 (4.11)	
Extra-skeletal Osteosarcoma	1 (0.79)	0 (0.00)	1 (1.37)	
Other	30 (23.62)	13 (24.07)	17 (23.29)	
Synovial sarcoma	9 (7.09)	5 (9.26)	4 (5.48)	
Ups	29 (22.83)	13 (24.07)	16 (21.92)	
Chemotherapy				
Anthracycline-based doublet	122 (96.06)	54 (100.00)	68 (93.15)	0.0715
Anthracycline monotherapy	5 (3.94)	0 (0.00)	5 (6.85)	
BMI				
<25	66 (51.97)	33 (61.11)	33 (45.21)	0.1109
≥25	61 (48.03)	21 (38.89)	40 (54.79)	

MPNST, malignant peripheral nerve sheath tumor; UPS, undifferentiated pleomorphic sarcoma; IQR, interquartile range; BMI, body mass index.

### 3.2 Toxicity analysis

**Figure 2:**
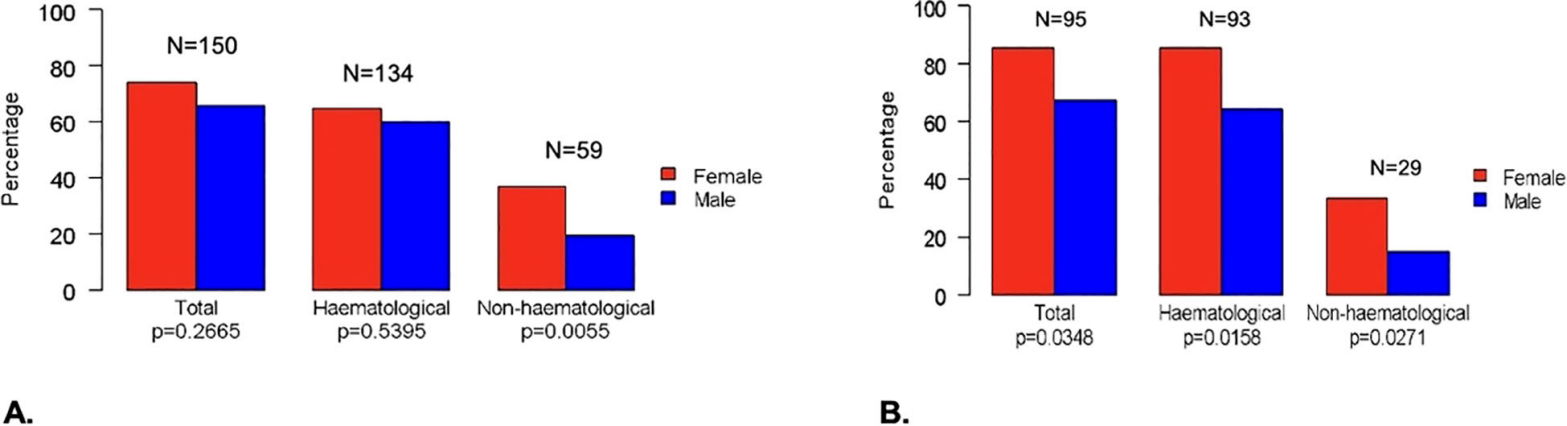
Differences between females and males for G3-G4 toxicity in all patients **(A)** and in Group 2 **(B)**.

**Figure 3:**
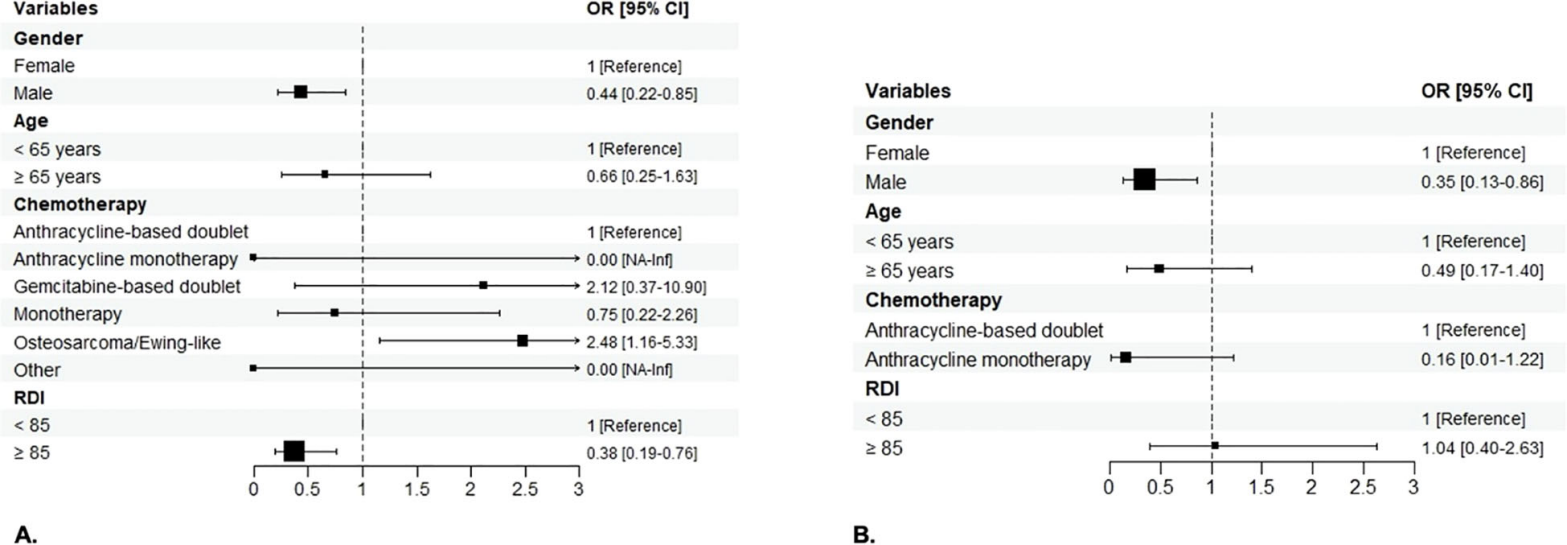
Forest plot of the association of patient sex, age, chemotherapy regimen, RDI and BMI with the risk of severe non-hematologic AEs in Group 1 **(A)** and hematologic AEs in Group 2 **(B)** on the multivariable analysis. The boxes indicate the OR, and the horizontal lines indicate the 95% CIs. AEs, adverse events; OR, odds ratio.

In Group 1, 150 (69.77%) patients experienced one or more severe (Grade ≥3) AEs, (females, 74 out of 100 [74%]; males, 76 out of 115 [66.09%]; p=0.2665). No G5 adverse events were observed. In Group 2, 95 (74.8%) patients experienced severe AEs (females, 46 out of 54 [85.19%]; males, 49 out of 73 [67.12%], p=0.0348) (**Figure 2**). In the univariable analysis, male sex was correlated with a lower risk of severe AEs. In particular, men were less likely to have non-hematologic toxicity compared to women in Group 1 ([OR]= 0.4, [95%CI 0.21 – 0.74], p< 0.0039), with a lower risk of G3-G4 overall toxicity ([OR]=0.36, [95%CI] 0.14 – 0.84; p=0.0235), both hematologic and non-hematologic AEs in those with STS treated with anthracycline-based chemotherapy (Group 2). A lower risk of severe non-hematologic AEs was seen in patients with RDI ≥ 85% compared to those with RDI <85% in Group 1 ([OR]= 0.38, [95%CI 0.2 – 0.71], p=0.0025), with similar results in Group 2 ([OR]=0.2, [95%CI] 0.08 – 0.47; p=0.0003). Chemotherapy regimens were confirmed to be significantly associated with toxicity, with anthracycline monotherapy (for both Groups) and single-agent chemotherapy (Group 1) being associated with a lower risk of severe AEs and osteosarcoma/Ewing-like perioperative therapy (Group 1) with a greater risk of severe toxicity compared to anthracycline-based doublets. Patients aged 65 years or older were less likely to experience G3-G4 overall toxicity in Groups. Only in Group 1 there was a lower likelihood of G3-G4 overall toxicity observed for BMI ≥ 25 ([OR]= 0.52; [95%CI 0.29 - 0.93]; p= 0.0290). On multivariable analysis, sex was confirmed as an independent factor influencing toxicity, with men having a lower risk of chemotherapy-related AEs. In particular, men were less likely to experience non-hematologic toxicity in Group 1 ([OR]= 0.44, [95%CI]; 0.22 – 0.85], p= 0.0151) (**Figure 3A**), with a lower risk of hematologic toxicity in Group 2 ([OR]= 0.35; [95%CI 0.13 – 0.86]; p= 0.0274)) (**Figure 3B**). A lower incidence of severe AEs was seen in patients with RDI ≥ 85% compared to those with RDI <85%. Chemotherapy regimens were confirmed to be significantly associated with toxicity, with anthracycline monotherapy, single-agent chemotherapies and gemcitabine-based doublets being associated with a lower risk of severe AEs and osteosarcoma/Ewing-like perioperative therapy being associated with a higher risk of toxicity compared with anthracycline-based doublets. No statistically significant differences in toxicity were seen between different age groups (< 65 vs ≥ 65 years) after adjusting for the other variables.

### 3.3 Analyses for relative dose intensity

**Figure 4:**
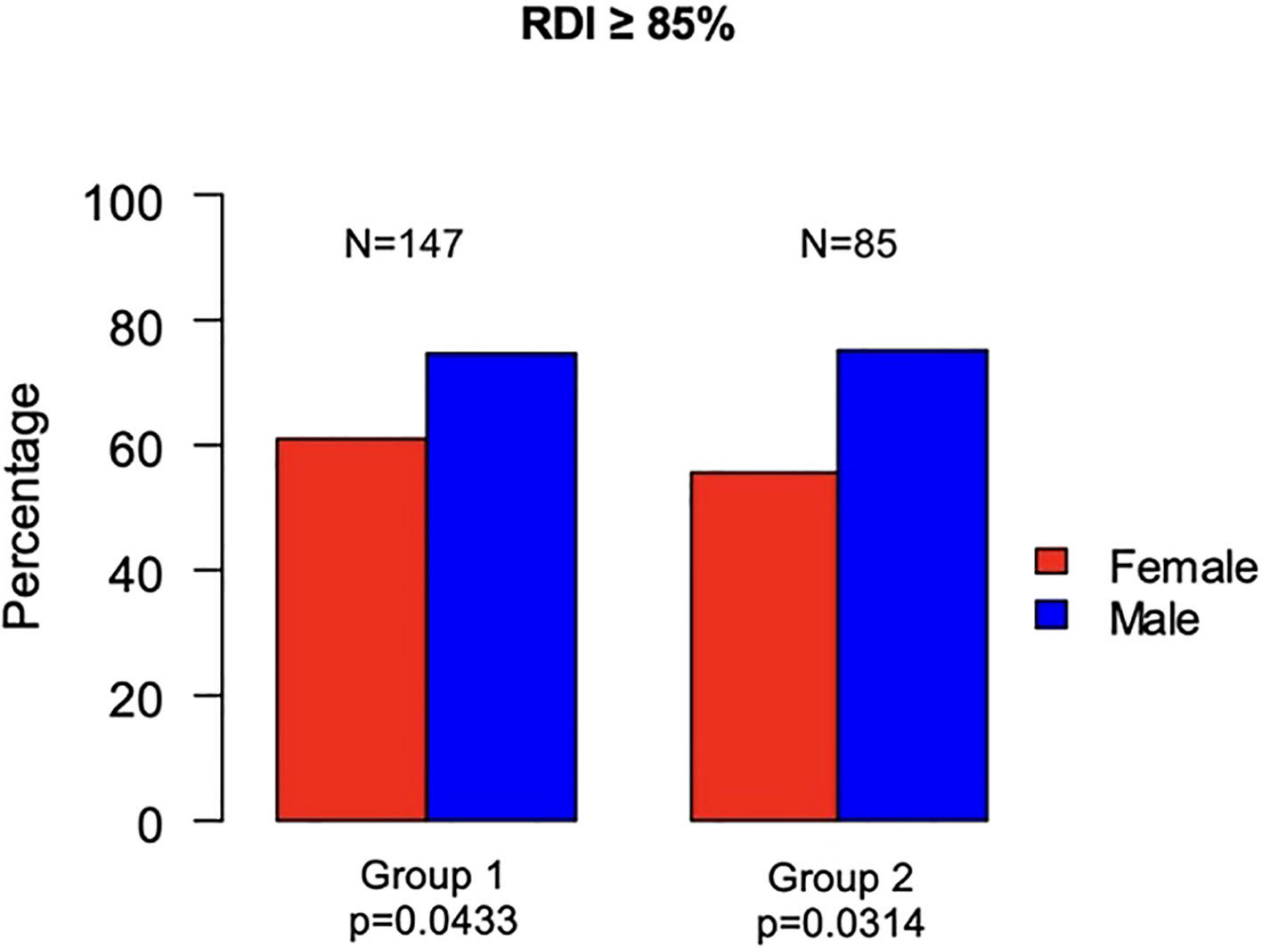
Differences between females and males for RDI.

**Figure 5:**
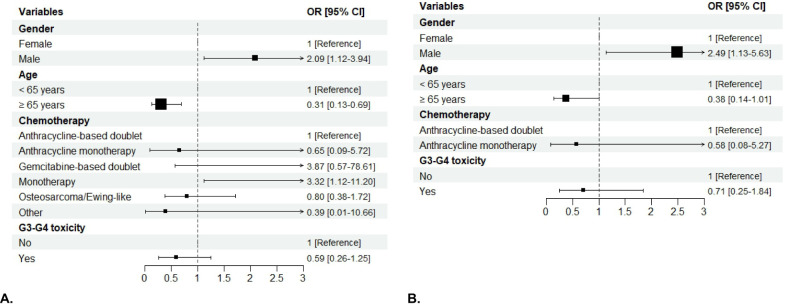
Forest plot of the association of patient sex, age, chemotherapy regimen, severe AEs and BMI with RDI in Group 1 **(A)** and in Group 2 **(B)** in the multivariable analysis. The boxes indicate the OR, and the horizontal lines indicate the 95% CIs. Boxes to the right of the vertical line (the line of equal odds) indicate increased risk of having RDI ≥ 85%, and to the left, lower risk. OR, odds ratio.

In Group 1, 147 (68.37%) patients had a RDI of chemotherapy ≥ 85% (men, 86 of 147 [58.5%]; women, 61 of 147 [41.5%]; p=0.0433). In Group 2, 85 (66.93%) patients had an RDI ≥ 85% (men, 55 of 85 [64.71%]; women, 30 of 85 [35.29%]; p=0.0314) (**Figure 4**). Both univariable and multivariable analysis confirmed that sex was significantly correlated with RDI, with males being more likely to have an RDI of chemotherapy ≥85% in both Group 1 ([OR]= 2.09; [95%CI 1.12 – 3.94]; p= 0.0207) and Group 2 ([OR]= 2.49; [95%CI 1.13 – 5.63]; p= 0.0250) (**Figure 5**).

Severe non-hematologic AEs were associated with a lower probability of having RDI ≥ 85% in both Groups, while patients experiencing G3-G4 overall toxicity were less likely to have RDI ≥ 85% only in Group 1 ([OR]= 0.38, [95%CI 0.2 – 0.71],p= 0.0025). In multivariable analysis, chemotherapy regimen was significantly associated with RDI, with single-agent chemotherapy being associated with a higher probability of having RDI ≥ 85% in Group 1 ([OR]= 3.32; [95%CI 1.12 – 11.20]; p= 0.0395). After adjusting for the other variables, age ≥ 65 years was significantly associated with lower RDI (p= 0.0049 for Group 1; p=0.05 for Group 2).

### 3.4 Survival analysis

In the entire group of patients, median follow up was 51.38 months (95%CI 35.76 - 62.34). Median DFS was not reached. Disease-free survival probability was 51.09% (95%CI 43.91 - 59.45), with a 3-year DFS probability of 54.08% (95%CI 47.19 - 61.99). Median OS was not reached; survival probability was 54.56% (95%CI 44.92 - 66.25), with a 3-year survival probability of 77.02% (95%CI 70.87 - 83.72). In Group 2, median follow-up was 57.96 months (95%CI 42.11 - 78.78). Median DFS and median OS were not reached. DFS probability was 57.83% (95%CI 48.83% - 68.49%), with a 3-year DFS probability of 59.35% (95%CI 50.51% - 69.75%). Survival probability was 56.09% (95%CI 44.07 - 71.39), with a 3-years survival probability of 79.96% (95%CI 72.32 - 88.41).

#### 3.4.1 Impact of RDI on survival outcomes

**Figure 6:**
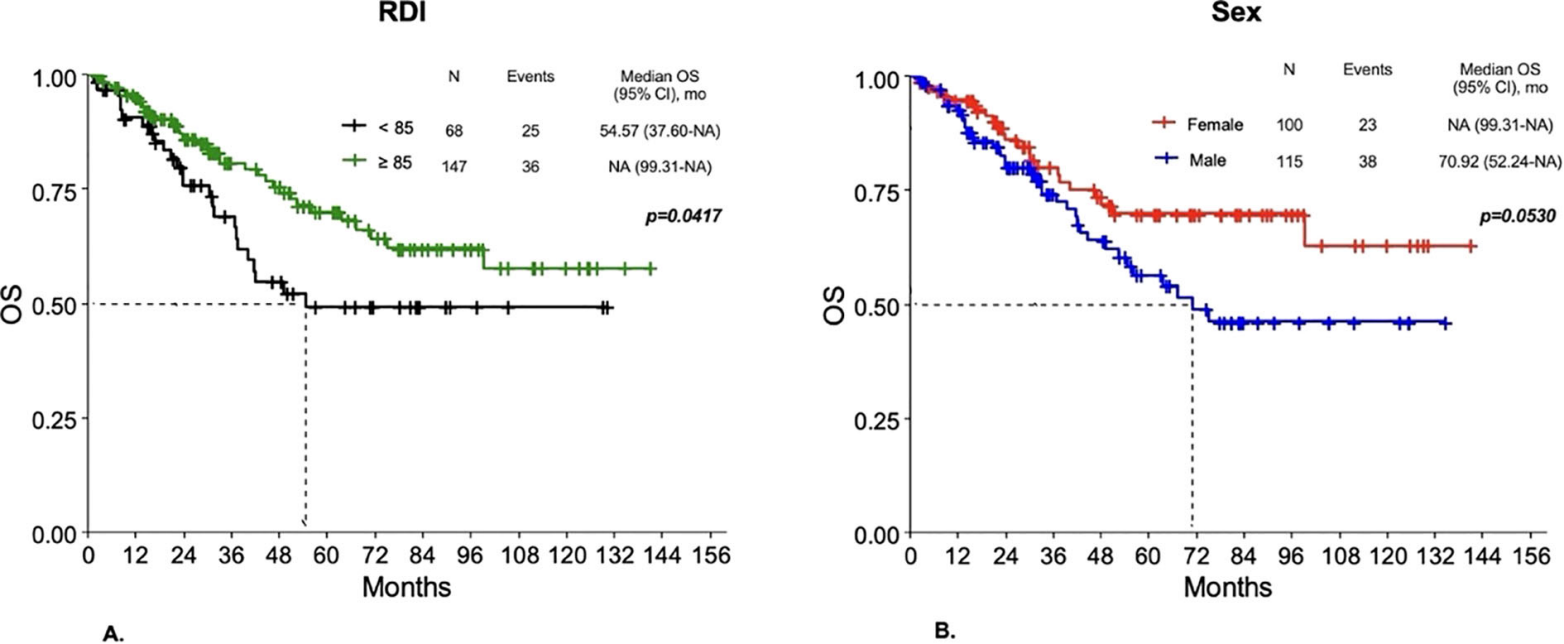
Overall survival **(A)** for RDI<85% vs RDI ≥85% and for females Vs males **(B)** in Group 1.

Overall, survival outcomes were better for patients with RDI ≥85% compared to those with a lower RDI. In Group 1, worse DFS was observed for RDI <85% compared to RDI ≥85% (p=0.0317), with a 3-year DFS probability of 45.2% for RDI <85% versus 58.14% for RDI ≥85% (p=0.0330). No statistically significant differences in DFS were observed according to sex or G3-G4 toxicity. Also as regards OS, a worse outcome was observed for patients with RDI <85% compared to those having RDI ≥85% (p=0.0417) (**Figure 6A**), with a 3-year survival probability of 69.02% for RDI <85% versus 80.69% for RDI ≥85% (p=0.0834). Women had a 3-year survival probability of 90.11%, compared to 74.23% of men (p=0.3162), with differences in OS not reaching statistical significance (p=0.053) (**Figure 6B**). No significant differences in OS were found according to G3-G4 toxicity. In Group 2, patients with RDI <85% had worse DFS compared to RDI ≥85% (p=0.0027), with a 3-year DFS probability of 40.28% for RDI <85% versus 69.06% for RDI ≥85% (p=0.0018). No statistically significant differences in DFS were observed according to sex or G3-G4 toxicity. Regarding OS, patients with RDI <85% had a 3-year survival probability of 70.64% compared to 85.07% of patients with RDI ≥85% (p=0.1169), with differences in OS not reaching statistical significance (p=0.0596). Differences for sex and G3-G4 toxicity were not statistically significant.

#### 3.4.2 Impact of sex and RDI on survival outcomes

**Figure 7:**
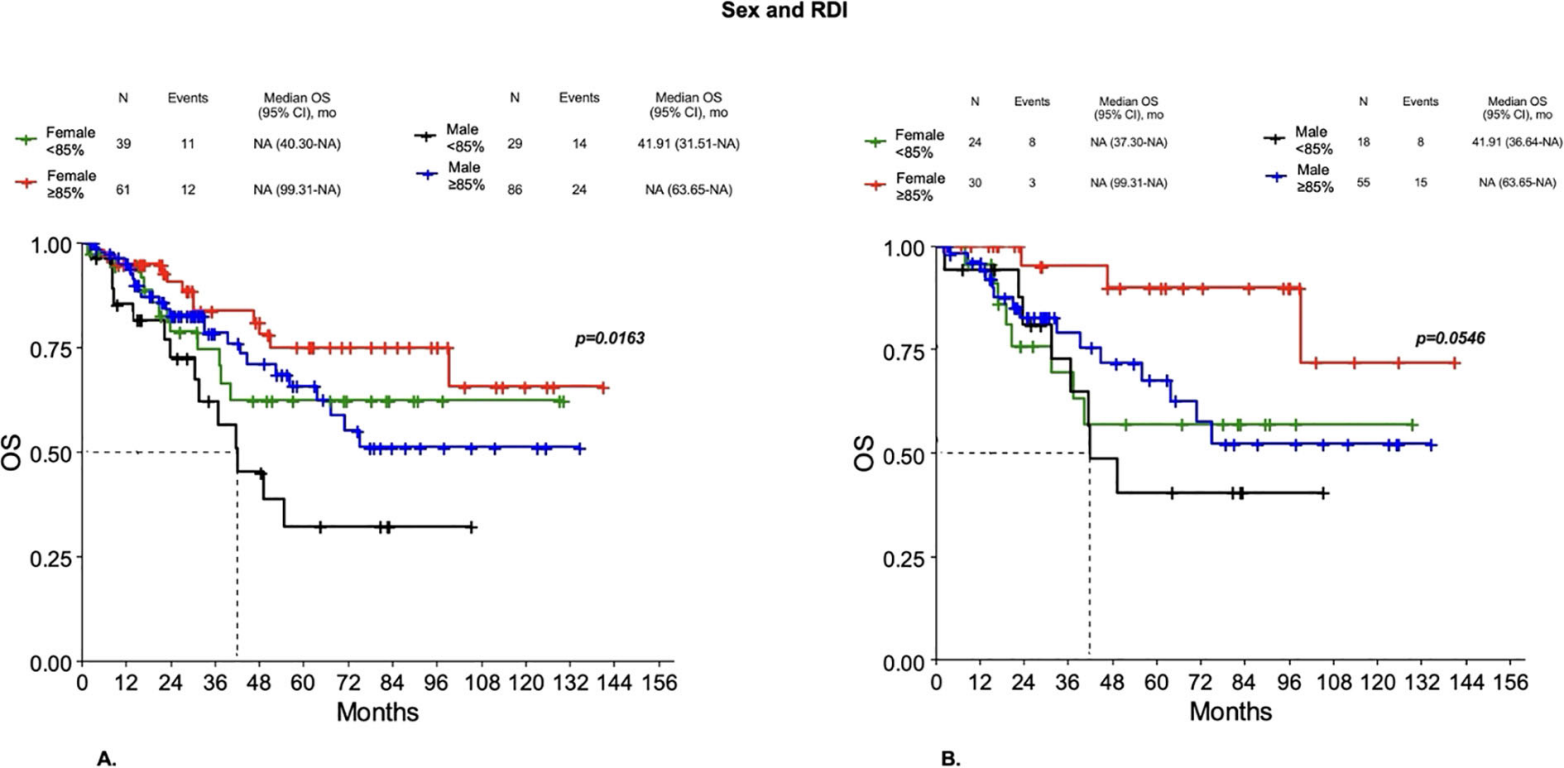
Overall survival for sex across different levels of RDI in Group 1 **(A)** and 2 **(B)**.

Even when they received the same dose reduction of chemotherapy, males showed worse outcomes than females. In particular, in Group 1 a worse DFS was observed for males with RDI < 85% compared to both females or males with RDI ≥85% and females with RDI <85% (p=0.0522). However, significant differences were observed for OS, with males with RDI <85% having worse OS compared to both females or males with RDI ≥85% and females with RDI < 85% (p=0.0163) (**Figure 7A**), with a 3-year survival probability of 62.12%, 83.66%, 78.4% and 74.71%, respectively (p=0.1752). In Group 2, worse DFS was observed for both males and females with RDI < 85% compared to both males or females with RDI ≥85% (p=0.0212), with a 3-year DFS probability of 36.36%, 42.48%, 68.43% and 69.48%, respectively (p=0.0155). A better OS was observed in females with RDI ≥85% compared to both males or females with RDI <85% and males with RDI ≥85%, but did not reach statistical significance (p=0.0546) (**Figure 7B**). Then, males, even when they received the same dose reduction of chemotherapy, showed worse outcomes than females.

## 4 Discussion

Our study showed that women were at higher risk of toxicity and were more likely to receive a lower RDI. There are several possible explanations for this. For instance, given average differences in body type, women might have received a higher relative dose, although we importantly included BMI to account for body type without observing a significant difference in toxicity across different BMI values. In addition, there might have been biases in the reporting or interpretation of AEs, because of possible sex-related differences in symptom perception (35, 36). However, in our study, objectively assessed AEs were also more common in women. Another possible explanation could be related to the type of administered chemotherapy. This may explain, at least in part, the higher risk of hematologic AEs observed in Group 2, whose patients were treated with highly myelotoxic anthracycline-based chemotherapy. Nevertheless, in our study, there was a fairly homogeneous distribution of the most toxic treatments between males and females. Furthermore, since older patients tend to receive lower doses of chemotherapy, we also included age as a variable in our analyses and no statistically significant differences in toxicity were seen between age groups after adjusting for other variables, probably due to confounding variables, most notably chemotherapy regimen. However, the number of women aged 65 years or older was similar to that of men. Sex differences in response to treatment have been reported in the literature in several disease settings, with female sex having been associated with an increased risk of AEs (24–28). This may be related to the fact that, until recently, women were excluded from clinical drug trials. Patient sex could influence both pharmacokinetics and pharmacodynamics, leading to differences in AEs across multiple classes of drugs (28). Metabolism and clearance of most chemotherapeutic agents are related to cytochrome P450 (CYP) isoenzymes (29), whose activity shows a wide inter-patient variation that is influenced by genetic polymorphisms and environmental factors (e.g. drugs or food). Age and sex also influence the activity of CYP isoenzymes (30). In particular, sex differences in the expression levels of drug-metabolizing enzymes and hormonal regulation of proteins involved in drug metabolism may play a role (31). In addition, several biological and psychosocial factors may contribute to women’s greater susceptibility to AEs (31). These include gut microbiota composition, sex hormone exposure, higher rates of polypharmacy in women, and, as noted above, differences in AE reporting (with women being more likely to report) (31). In addition, women seem to have a greater risk of overdose due to lower volume of distribution, higher body fat percentage, and slower xenobiotic clearance (30). Several studies also show that lifestyle factors (e.g., tobacco, alcohol, diet, physical inactivity), which are known to have a direct effect on drug response, differ greatly between men and women (32, 33). However, dosage recommendations for anticancer drugs are not sex-specific (34–36). As for sarcomas, the few available data support the hypothesis that sex may influence drug toxicity. In patients with Ewing sarcoma enrolled in the ISG/SSG (Italian Sarcoma Group/Scandinavian Sarcoma Group) III protocol and receiving treatment based on vincristine, doxorubicin, cyclophosphamide, ifosfamide, dactinomycin and etoposide, a lower risk of G4 leukopenia and thrombocytopenia, febrile neutropenia, hospitalization, and red blood cell transfusions was observed in males (37). In the Euro-E.W.I.N.G.99-R1 randomized trial comparing the efficacy of VAC (vincristine, dactynomycin, cyclophosphamide) vs VAI (vincristine, dactynomycin, ifosfamide) chemotherapy as maintenance treatment for localized Ewing sarcoma, VAC was associated with worse event-free survival (EFS) than VAI in males, whereas EFS was slightly better in females treated with VAC than VAI (38). Based on these premises, a meta-analysis on the interaction between alkylating agents and gender (MAIAGE) of randomized trials comparing cyclophosphamide and ifosfamide was performed. However, it did not confirm the hypothesis of heterogeneity of the efficacy and toxicity of alkylating agents between males and females (39). In addition, an exploratory study investigated the impact of sex on the efficacy and acute toxicity of alkylating agent-based chemotherapy in patients treated in the Euro-E.W.I.N.G.99-R1 trial. It showed that more females than males experienced severe toxicities (e.g., hematologic AEs, infections, renal toxicity), while the effect of VAC vs VAI treatment on the risk of toxicity did not differ significantly between males and females (40). Consistent with the higher risk of toxicity associated with female sex, women in our study population were more likely to have a lower RDI compared to men (p=0.0001). In addition, non-hematologic AEs were the type of toxicity most frequently associated with lower RDI levels. This is likely due to the fact that hematologic AEs can be mainly prevented and/or managed compared to non-hematologic AEs, for example by the use of granulocyte-colony stimulating factors (G-CSF) or by recourse to red blood cell or platelet transfusion. Moreover, management of toxicities may contribute to maintaining a higher RDI and then benefit patient survival. Indeed, maintaining higher levels of RDI has been reported to be associated with improved survival in breast cancer and other solid tumors (11, 12, 41–43). In particular, in patients with breast cancer treated with anthracycline (epirubicin 60 to 90 mg/m2 or doxorubicin 60 mg/m2) based therapy, optimizing RDI above 85% has been shown to prolong overall survival (11, 41). In addition, in a study investigating the impact of dose delays and/or reductions in patients with solid tumors receiving adjuvant or neoadjuvant chemotherapy, reduced RDI were lowest among patients with breast cancer compared to other tumors. As observed in our study, neutropenia was the most common toxicity-related reason for dose delays and reductions, followed by anemia, thrombocytopenia, fatigue, nausea/vomiting and mucositis (18). The doses of anthracyclines used in sarcomas (doxorubicin 75 mg/m2 or epirubicin 120 mg/m2) are higher compared to those used for breast cancer (epirubicin 60 to 90 mg/m2 or doxorubicin 60 mg/m2). This could explain the lower RDI observed in females treated with anthracyclines in our study compared to that reported in studies on women treated with anthracyclines for breast cancer. Moreover, as observed for breast cancer and other solid tumors, in our study, higher RDI has also been reported to be associated with improved survival, supporting the hypothesis that the RDI is a predictor of outcome, regardless of tumor histology and the standard doses of chemotherapy. Interestingly, in our study, the more frequent dose reductions in women compared to men have not led to worse survival outcomes in women. Several studies have shown better outcomes in females treated for non-sex-related cancers (44–48). A retrospective analysis of patients with Ewing sarcoma showed that female sex was associated with a survival benefit only in Caucasian patients (47). Sleijfer et al. retrospectively analyzed data from patients with advanced STS who received first-line ifosfamide-containing chemotherapy. In addition to good performance status, non-metastatic disease, extremity primary tumor and low grade, female sex was also found to be an independent favorable prognostic factor for OS (48). Furthermore, Buja et al. retrospectively analyzed epidemiological data of patients with STS (49). No significant sex differences were found in short-term mortality or according to clinicopathological profile, except for cancer site, with more retroperitoneal involvement in females and more limb or head/neck involvement in males (49–51). Moreover, sex-based toxicity and survival differences could be biologically underpinned by differential gene expression and chemosensitivity pathways (52). In a study focused on myxofibrosarcoma (MFS) and undifferentiated pleomorphic sarcoma (UPS), Vanni et al. identified the down-regulation of immunoglobulin genes (IGKV2D-30, IGKV1D-13, IGHV3-72, IGLV3-10, IGHV1-69-2, IGKV3D-15) in patient-derived primary cultures that responded to anthracycline treatment compared to non-responder cultures. They also found an up-regulation of doxorubicin metabolic processes in MFS compared to UPS (52). Nevertheless, it remains unclear why male sex puts patients at risk for decreased survival. Differences in pharmacokinetics and pharmacodynamics, genetic variations, as well as hormonal differences and social aspects could play a role. However, we cannot exclude that other possible mechanisms, which are still unknown, may also be involved. In our study, there might have been biases in the selection of patients (53, 54), both regarding the specific histotype, with some being more aggressive than others, and the distribution between females and males of the more aggressive sarcomas. Nevertheless, our results are robust due to the breadth of the data and the large sample size. Indeed, in our series we obtained results consistent with the available epidemiologic data , both in terms of the distribution of histotypes among soft tissue and/or bone sarcomas, and in terms of the male-to-female ratio for each histotype. However, our study has limitations. First, the retrospective observational nature of its design. As in other retrospective studies, the strategy was to collect the maximum number of informative cases to ensure a statistically adequate sample size. The large Groups obtained proved sufficient to detect the strong association of most of the variables considered, but we cannot exclude that minor associations were missed. In addition, although we addressed potential confounding by including only untreated patients with localized sarcomas and/or by statistically adjusting for age, chemotherapy regimen and BMI, some of the associations between sex and toxicity may still be attributable to confounding factors such as comorbidity, worse performance status or tumor site. In addition, reporting of AE data may be subject to misclassification, particularly when CTCAE criteria are unable to classify subtle symptoms. For this reason, our primary analyses included only severe AEs, which are more easily recognized. Also, symptomatic AEs were not actually reported by the patients themselves. Despite these limitations, this study has important strengths. To the authors’ knowledge, this is the first study to focus on the impact of sex on the toxicity and efficacy of anticancer therapies in patients treated for both soft tissue and bone sarcomas in the perioperative setting.

## 5 Conclusions

The greater risk of severe chemotherapy-related acute toxicity and the lower RDI observed in women suggest that they respond differently from men to pharmacological treatment. Moreover, the improved survival outcomes associated with higher RDI indicate that management of toxicities may contribute to maintaining higher RDI and benefit survival. Although females received overall lower RDI of chemotherapy, survival outcomes were better for them compared to males. According to the literature, a higher risk of toxicity as well as better outcomes have been observed in females treated for non-sex-related cancers, including sarcomas. Higher RDI has also been reported to be associated with improved survival for several solid tumors. Historically, women have often been excluded from clinical trials. As a result, the impact of sex on both toxicity and clinical outcomes has long been underestimated. Although women are now systematically included in clinical trials, patient sex is rarely taken into account in clinical research. The role of BMI in the differences between females and males observed in patients undergoing chemotherapy for sarcoma remains to be evaluated. Thus, in the era of precision medicine, there remains an unmet need to gain a deeper understanding of the underlying processes behind sex differences and the weight they may have in clinical decision making. In this context, future studies should aim to optimize drug dosing by sex, with the ultimate goal of extending therapeutic benefit while limiting toxicity, especially for women.

## Data Availability

The raw data supporting the conclusions of this article will be made available by the authors, without undue reservation.

## References

[1] Wagner AD. Sex differences in cancer chemotherapy effects, and why we need to reconsider BSA-based dosing of chemotherapy. ESMO Open. (2020) 5:e000770. doi: 10.1136/esmoopen-2020-000770 32967917 PMC7513669

[2] Baraibar I Ros J Saoudi N Salvà F García A Castells MR. Sex and gender perspectives in colorectal cancer. ESMO Open. (2023) 8:101204. doi: 10.1016/j.esmoop.2023.101204 37018873 PMC10163160

[3] Sung H Ferlay J Siegel RL Laversanne M Soerjomataram I Jemal A. Global cancer statistics 2020: GLOBOCAN estimates of incidence and mortality worldwide for 36 cancers in 185 countries. CA Cancer J Clin. (2021) 71:209–49. doi: 10.3322/caac.21660 33538338

[4] Clocchiatti A Cora E Zhang Y Dotto GP. Sexual dimorphism in cancer. Nat Rev Cancer. (2016) 16:330–9. doi: 10.1038/nrc.2016.30 27079803

[5] Li H Jiang W Liu S Yang M Chen S Pan Y. Connecting the mechanisms of tumor sex differences with cancer therapy. Mol Cell Biochem. (2023). doi: 10.1007/s11010-023-04723-1 37027097

[6] Rakshith HT Lohita S Rebello AP Goudanavar PS Raghavendra Naveen N. Sex differences in drug effects and/or toxicity in oncology. Curr Res Pharmacol Drug Discov. (2023) 4:100152. doi: 10.1016/j.crphar.2022.100152 36714036 PMC9881040

[7] Unger JM Vaidya R Albain KS LeBlanc M Minasian LM Gotay CC. Sex differences in risk of severe adverse events in patients receiving immunotherapy, targeted therapy, or chemotherapy in cancer clinical trials. J Clin Oncol. (2022) 40:1474–86. doi: 10.1200/JCO.21.02377 PMC9061143 35119908

[8] General considerations for the clinical evaluation of drugs. United States: Food and Drug Administration. Rockville, Md.: U. S. Dept. of Health Education, and Welfare, Public Health Service, Food and Drug Administration. Washington: for sale by the Supt. of Docs., U. S. Govt. Print. Off. Available online at: https://www.fda.gov/regulatory-information/search-fdaguidance-documents/generalconsiderations-clinical-evaluation-drugs.

[9] Investigational new drug applications; amendment to clinical hold regulations for products intended for life-threatening diseases and conditions; final rule. Fed Regist. (2000) 65(106):34963–71. 11010726

[10] Nielson CM Bylsma LC Fryzek JP Saad HA Crawford J. Relative dose intensity of chemotherapy and survival in patients with advanced stage solid tumor cancer: A systematic review and meta-analysis. Oncologist. (2021) 26:e1609–18. doi: 10.1002/onco.13822 PMC8417866 33973301

[11] Loibl S Skacel T Nekljudova V Lück HJ Schwenkglenks M Brodowicz T. Evaluating the impact of Relative Total Dose Intensity (RTDI) on patients’ short and long-term outcome in taxane- and anthracycline-based chemotherapy of metastatic breast cancer- a pooled analysis. BMC Cancer. (2011) 11:131. doi: 10.1186/1471-2407-11-131 21486442 PMC3083375

[12] Hanna RK Poniewierski MS Laskey RA Lopez MA Shafer A Van Le L. Predictors of reduced relative dose intensity and its relationship to mortality in women receiving multi-agent chemotherapy for epithelial ovarian cancer. Gynecol Oncol. (2013) 129:74–80. doi: 10.1016/j.ygyno.2012.12.017 23262376

[13] Havrilesky LJ Reiner M Morrow PK Watson H Crawford J. A review of relative dose intensity and survival in patients with metastatic solid tumors. Crit Rev Oncol Hematol. (2015) 93:203–10. doi: 10.1016/j.critrevonc.2014.10.006 25459671

[14] Shayne M Harvey RD Lyman GH. Prophylaxis and treatment strategies for optimizing chemotherapy relative dose intensity. Expert Rev Anticancer Ther. (2021) 21:1145–59. doi: 10.1080/14737140.2021.1941891 34114525

[15] Bonadonna G Valagussa P. Dose-response effect of adjuvant chemotherapy in breast cancer. N Engl J Med. (1981) 304:10–5. doi: 10.1056/NEJM198101013040103 7432433

[16] Bonadonna G Valagussa P Moliterni A Zambetti M Brambilla C. Adjuvant cyclophosphamide, methotrexate, and fluorouracil in node-positive breast cancer: the results of 20 years of follow-up. N Engl J Med. (1995) 332:901–6. doi: 10.1056/NEJM199504063321401 7877646

[17] Zhang L Yu Q Wu XC Hsieh MC Loch M Chen VW. Impact of chemotherapy relative dose intensity on cause-specific and overall survival for stage I-III breast cancer: ER+/PR+, HER2- vs. triple-negative. Breast Cancer Res Treat. (2018) 169:175–87. doi: 10.1007/s10549-017-4646-1 PMC6190707 29368311

[18] Denduluri N Patt DA Wang Y Bhor M Li X Favret AM. Dose delays, dose reductions, and relative dose intensity in patients with cancer who received adjuvant or neoadjuvant chemotherapy in community oncology practices. J Natl Compr Canc Netw. (2015) 13:1383–93. doi: 10.6004/jnccn.2015.0166 26553767

[19] Citron ML. Dose-dense chemotherapy: principles, clinical results and future perspectives. Breast Care (Basel). (2008) 3:251–5. doi: 10.1159/000148914 PMC2974980 21076605

[20] Womer RB West DC Krailo MD Dickman PS Pawel BR Grier HE. Randomized controlled trial of interval-compressed chemotherapy for the treatment of localized Ewing sarcoma: a report from the Children’s Oncology Group. J Clin Oncol. (2012) 30:4148–54. doi: 10.1200/JCO.2011.41.5703 PMC3494838 23091096

[21] Lewis IJ Weeden S Machin D Stark D Craft AW. Received dose and dose-intensity of chemotherapy and outcome in nonmetastatic extremity osteosarcoma. J Clin Oncol. (2000) 18:4028–37. doi: 10.1200/JCO.2000.18.24.4028 11118463

[22] Bui-Nguyen B Ray-Coquard I Chevreau C Penel N Bay JO Coindre JM. High-dose chemotherapy consolidation for chemosensitive advanced soft tissue sarcoma patients: an open-label, randomized controlled trial. Ann Oncol. (2012) 23:777–84. doi: 10.1093/annonc/mdr282 21652583

[23] National Cancer Institute. CTEP: Cancer Therapy Evaluation Program. (2021). Available online at: https://ctep.cancer.gov/protocoldevelopment/electronic_applications/ctc.htm (Accessed April 19, 2022).

[24] Chansky K Benedetti J Macdonald JS. Differences in toxicity between men and women treated with 5-fluorouracil therapy for colorectal carcinoma. Cancer. (2005) 103(6):1165–71. doi: 10.1002/cncr.20878 15693031

[25] Gotay CC Phillips PH Cheson BD. Male-female differences in the impact of cancer therapy. Oncol (Williston Park). (1993) 7(2):67–74. 8439470

[26] Sloan JA Goldberg RM Sargent DJ Vargas-Chanes D Nair S Cha SS. Women experience greater toxicity with fluorouracil-based chemotherapy for colorectal cancer. J Clin Oncol. (2002) 20:1491–8. doi: 10.1200/JCO.2002.20.6.1491 11896096

[27] Rademaker M. Do women have more adverse drug reactions? Am J Clin Dermatol. (2001) 2:349–51. doi: 10.2165/00128071-200102060-00001 11770389

[28] Zucker I Prendergast BJ. Sex differences in pharmacokinetics predict adverse drug reactions in women. Biol Sex Differ. (2020) 11:32. doi: 10.1186/s13293-020-00308-5 32503637 PMC7275616

[29] Scripture CD Sparreboom A Figg WD. Modulation of cytochrome P450 activity: implications for cancer therapy. Lancet Oncol. (2005) 6:780–9. doi: 10.1016/S1470-2045(05)70388-0 16198984

[30] Bebia Z Buch SC Wilson JW Frye RF Romkes M Cecchetti A. Bioequivalence revisited: influence of age and sex on CYP enzymes. Clin Pharmacol Ther. (2004) 76:618–27. doi: 10.1016/j.clpt.2004.08.021 15592333

[31] Özdemir BC Gerard CL Espinosa da Silva C. Sex and gender differences in anticancer treatment toxicity: A call for revisiting drug dosing in oncology. Endocrinology. (2022) 163:bqac058. doi: 10.1210/endocr/bqac058 35560216 PMC9113364

[32] Gusella M Crepaldi G Barile C Bononi A Menon D Toso S. Pharmacokinetic and demographic markers of 5-fluorouracil toxicity in 181 patients on adjuvant therapy for colorectal cancer. Ann Oncol. (2006) 17:1656–60. doi: 10.1093/annonc/mdl284 16968871

[33] Dobbs NA Twelves CJ Gillies H James CA Harper PG Rubens RD. Gender affects doxorubicin pharmacokinetics in patients with normal liver biochemistry. Cancer Chemother Pharmacol. (1995) 36:473–6. doi: 10.1007/BF00685796 7554038

[34] Lu JF Bruno R Eppler S Novotny W Lum B Gaudreault J. Clinical pharmacokinetics of bevacizumab in patients with solid tumors. Cancer Chemother Pharmacol. (2008) 62:779–86. doi: 10.1007/s00280-007-0664-8 18205003

[35] Krishnamurthy P Schuetz JD. Role of ABCG2/BCRP in biology and medicine. Annu Rev Pharmacol Toxicol. (2006) 46:381–410. doi: 10.1146/annurev.pharmtox.46.120604.141238 16402910

[36] Merino G van Herwaarden AE Wagenaar E Jonker JW Schinkel AH. Sex-dependent expression and activity of the ATP-binding cassette transporter breast cancer resistance protein (BCRP/ABCG2) in liver. Mol Pharmacol. (2005) 67:1765–71. doi: 10.1124/mol.105.011080 15722455

[37] Paioli A Luksch R Fagioli F Tamburini A Cesari M Palmerini E. Chemotherapy-related toxicity in patients with non-metastatic Ewing sarcoma: influence of sex and age. J Chemother. (2014) 26:49–56. doi: 10.1179/1973947813Y.0000000103 24091100

[38] Le Deley MC Paulussen M Lewis I Brennan B Ranft A Whelan J. Cyclophosphamide compared with ifosfamide in consolidation treatment of standard-risk Ewing sarcoma: results of the randomized noninferiority Euro-EWING99-R1 trial. J Clin Oncol. (2014) 32:2440–8. doi: 10.1200/JCO.2013.54.4833 24982464

[39] Fresneau B Hackshaw A Hawkins DS Paulussen M Anderson JR Judson I. Investigating the heterogeneity of alkylating agents’ efficacy and toxicity between sexes: A systematic review and meta-analysis of randomized trials comparing cyclophosphamide and ifosfamide (MAIAGE study). Pediatr Blood Cancer. (2017) 64. doi: 10.1002/pbc.26457 28111876

[40] van den Berg H Paulussen M Le Teuff G Judson I Gelderblom H Dirksen U. Impact of gender on efficacy and acute toxicity of alkylating agent -based chemotherapy in Ewing sarcoma: secondary analysis of the Euro-Ewing99-R1 trial. Eur J Cancer. (2015) 51:2453–64. doi: 10.1016/j.ejca.2015.06.123 26271204

[41] Abdel-Rahman O. Outcomes of early-stage breast cancer patients treated with sequential anthracyclines-taxanes in relationship to relative dosing intensity: a secondary analysis of a randomized controlled trial. Clin Transl Oncol. (2019) 21:239–45. doi: 10.1007/s12094-018-1915-3 29956074

[42] Aspinall SL Good CB Zhao X Cunningham FE Heron BB Geraci M. Adjuvant chemotherapy for stage III colon cancer: relative dose intensity and survival among veterans. BMC Cancer. (2015) 15:62. doi: 10.1186/s12885-015-1038-y 25884851 PMC4352567

[43] Yabusaki N Fujii T Yamada S Murotani K Sugimoto H Kanda M. The significance of relative dose intensity in adjuvant chemotherapy of pancreatic ductal adenocarcinoma-including the analysis of clinicopathological factors influencing relative dose intensity. Med (Baltimore). (2016) 95:e4282. doi: 10.1097/MD.0000000000004282 PMC5265784 27442667

[44] Crocetti E Mallone S Robsahm TE Gavin A Agius D Ardanaz E. Survival of patients with skin melanoma in Europe increases further: Results of the EUROCARE-5 study. Eur J Cancer. (2015) 51:2179–90. doi: 10.1016/j.ejca.2015.07.039 26421821

[45] Yamamoto H Sekine I Yamada K Nokihara H Yamamoto N Kunitoh H. Gender differences in treatment outcomes among patients with non-small cell lung cancer given a combination of carboplatin and paclitaxel. Oncology. (2008) 75:169–74. doi: 10.1159/000159268 18827494

[46] Lorence J Bencomo T White H Rickertsen C Massey S Singleton K. NCOG-69. Sex differences in glioblastoma patient survival as a function of extent of surgical resection and cycles of adjuvant temozolomide during standard-of-care regimens. Neuro Oncol. (2020) 22:ii144–5. doi: 10.1093/neuonc/noaa215.607

[47] Jawad MU Cheung MC Min ES Schneiderbauer MM Koniaris LG Scully SP. Ewing sarcoma demonstrates racial disparities in incidence-related and sex-related differences in outcome: an analysis of 1631 cases from the SEER database, 1973-2005. Cancer. (2009) 115:3526–36. doi: 10.1002/cncr.24388 19548262

[48] Sleijfer S Ouali M van Glabbeke M Krarup-Hansen A Rodenhuis S Le Cesne A. Prognostic and predictive factors for outcome to first-line ifosfamide-containing chemotherapy for adult patients with advanced soft tissue sarcomas: an exploratory, retrospective analysis on large series from the European Organization for Research and Treatment of Cancer-Soft Tissue and Bone Sarcoma Group (EORTC-STBSG). Eur J Cancer. (2010) 46:72–83. doi: 10.1016/j.ejca.2009.09.022 19853437

[49] Buja A Rugge M Tropea S Cozzolino C Formaro CM Grotto G. Sex differences in soft tissue sarcoma: incidence, clinicopathological profile, survival, and costs. J Womens Health (Larchmt). (2023) 32:1257–64. doi: 10.1089/jwh.2023.0019 PMC10621658 37819711

[50] Edwards D Voronina A Attwood K Grand’Maison A. Association between occupational exposures and sarcoma incidence and mortality: systematic review and meta-analysis. Syst Rev. (2021) 10:231. doi: 10.1186/s13643-021-01769-4 34389054 PMC8364027

[51] Wingren G Fredrikson M Brage HN Nordenskjöld B Axelson O. Soft tissue sarcoma and occupational exposures. Cancer. (1990) 66:806–11. doi: 10.1002/1097-0142(19900815)66:4<806::aid-cncr2820660435>3.0.co;2-u 2386907

[52] Vanni S Fausti V Fonzi E Liverani C Miserocchi G Spadazzi C. Unveiling the genomic basis of chemosensitivity in sarcomas of the extremities: an integrated approach for an unmet clinical need. Int J Mol Sci. (2023) 24:6926. doi: 10.3390/ijms24086926 37108089 PMC10138892

[53] Howlader N Noone AM Krapcho M. SEER cancer statistics review, 1975-2012. Based on November 2014 SEER data submission, posted to the SEER web site (2015). Available online at: http://seer.cancer.gov/csr/1975_2012/ (Accessed December 22, 2015).

[54] Strauss SJ Frezza AM Abecassis N Bajpai J Bauer S Biagini R. ESMO Guidelines Committee, EURACAN, GENTURIS and ERN PaedCan. Electronic address: clinicalguidelines@esmo.org. Bone sarcomas: ESMO-EURACAN-GENTURIS-ERN PaedCan Clinical Practice Guideline for diagnosis, treatment and follow-up. Ann Oncol. (2021) 32:1520–36. doi: 10.1016/j.annonc.2021.08.1995 34500044

